# Caregiver Screening for Relapse Among Children Recently Recovered From Severe Acute Malnutrition: A Randomized Controlled Feasibility Trial

**DOI:** 10.1111/mcn.70160

**Published:** 2026-01-22

**Authors:** Mamadou Bountogo, Clarisse Dah, Moussa Ouédraogo, Fanta Zerbo, Idrissa Kouanda, Elodie Lebas, Ian Fetterman, Jessica La Mons, Hadley Burroughs, Benjamin F. Arnold, Ali Sié, Catherine E. Oldenburg

**Affiliations:** ^1^ Centre de Recherche en Santé de Nouna Nouna Burkina Faso; ^2^ Francis I Proctor Foundation University of California San Francisco California USA; ^3^ Department of Ophthalmology University of California San Francisco California USA; ^4^ Department of Epidemiology & Biostatistics University of California San Francisco California USA

**Keywords:** mid‐upper arm circumference, randomized controlled trial, screening, severe acute malnutrition

## Abstract

Relapse to acute malnutrition after recovery from severe acute malnutrition (SAM) is common. However, most programmatic resources are devoted to the acute phase of recovery, and fewer interventions are available for children recently discharged from outpatient nutritional programs. We evaluated the feasibility of training caregivers to screen for relapse using mid‐upper arm circumference (MUAC) tapes for reducing time to detection of relapse among children recently recovered from SAM in Burkina Faso. Caregiver‐child dyads were enrolled and randomized in a 1:1 fashion to either caregiver MUAC screening or local standard of care (SOC), which consists of monthly clinic‐based follow‐up visits for 3 months following discharge. In the MUAC screening group, caregivers were trained on how to use a standard MUAC tape and asked to screen their child weekly with a provided MUAC tape for the 6‐month duration of the study. The primary outcome was time to relapse detection, defined as MUAC < 12.5 cm and/or weight‐for‐height Z‐score < −2. Secondary outcomes included hospitalization and/or death over the 6‐month study period. Of 200 caregiver‐child dyads enrolled in the trial, 99 were randomized to the MUAC screening group and 101 to the SOC group. By 6 months after enrollment, the hazard of relapse detection was lower in the MUAC screening group compared to the SOC group (hazard ratio, HR, 0.65, 95% confidence interval, CI, 0.38–1.12). Fewer hospitalizations and/or deaths occurred in the MUAC screening group compared to the SOC group (MUAC: 3%; SOC: 14%, risk ratio 0.23, 95% CI, 0.07–0.79). Training caregivers to screen for relapse after recovery from SAM was feasible and may lead to modestly reduced time to detection of relapse, suggesting a full‐scale trial is warranted.

**Trial Registration:** This study was prospectively registered on clinicaltrials.gov (NCT05932992, registered June 27, 2023).

## Summary

- Relapse to acute malnutrition after recovery from severe acute malnutrition is common, but most interventions are focused on the acute phase of recovery.
- Mid‐upper arm circumference (MUAC) measurements are used for screening for acute malnutrition and have been used in community‐based settings by caregivers to assess their children for acute malnutrition.
- Children whose caregivers had been trained to screen for relapse using MUAC tapes had modestly improved time to detection of relapse detection, but the overall primary clinical endpoint was not statistically significant.
- Equipping caregivers with MUAC tapes may lead to improved awareness of a child's health status or earlier detection of relapse which may improve clinical outcomes.

## 1 Introduction

Severe acute malnutrition (SAM) is a severe form of wasting that affects millions of children under 5 years of age each year (Black et al. 2013). Nutritional programs provide community‐based outpatient treatment for SAM for children without complications requiring hospitalization (World Health Organization. 2023). These programs focus on acute recovery, including provision of ready‐to‐use therapeutic food (RUTF), and prioritize rapid weight gain to prevent mortality and acute morbidity. Outpatient provision of care has increased access to nutritional program and improved short‐term outcomes for children. However, the risk of relapse is high in children who have recently recovered from SAM (King et al. 2025; Stobaugh et al. 2019). Substantially less evidence exists for prevention of relapse after recovery compared to treatment of the acute phase of SAM.

“Nutritional recovery” is based on achievement of arbitrary thresholds, including achievement of a weight‐for‐height Z‐score (WHZ) of ≥ −2 and/or mid‐upper arm circumference MUAC) of ≥ 12.5 cm, depending on which criteria were used for admission to the nutritional program (these criteria have been updated in the 2023 World Health Organization guidelines recommending that both WHZ and MUAC thresholds be met regardless of admission criteria^2^). While these children have gained enough weight to meet criteria for recovery, they may still have significant anthropometric deficits or other risk factors that could increase likelihood of relapse. Guidelines for the management of children post‐recovery are limited and supported by a limited evidence base. In Burkina Faso, guidelines include monthly surveillance for 3 months post‐discharge. For children with SAM, worse anthropometric deficits at admission to the nutritional program led to worse outcomes, including increased risk of relapse (Stobaugh et al. 2018; Dah et al. 2022). Earlier identification of relapse may be beneficial for children, as they can be re‐engaged more quickly with the nutritional program to reduce risk of mortality and morbidity due to malnutrition (Ahmed et al. 2025).

In community‐based settings, mothers have been successfully trained to screen for new‐onset global acute malnutrition (GAM) using standard color‐coded MUAC tapes (Blackwell et al. 2015; Alé et al. 2016). MUAC‐based screening for GAM has been used at large scale by community health workers in many settings. MUAC tapes are easy to use, do not require major equipment, and do not require those using them to be literate, as they identify children with acute malnutrition based on color coding. Training of caregivers to screen for acute malnutrition using MUAC tapes (e.g. “family MUAC”) has been piloted in community‐based management of acute malnutrition programs in 16 countries in Africa (Majiwa et al. 2024; Bliss et al. 2018). Compared to community health worker screening, family MUAC may lead to earlier detection of SAM (Tamirat et al. 2025). Here, we report the results of a randomized controlled trial designed to evaluate the feasibility of training caregivers of children who recently recovered from SAM to screen for signs of relapse. A feasibility trial was conducted to assess both acceptability and preliminary effectiveness of the intervention. We hypothesized that caregiver screening using MUAC tapes would be feasible and would lead to improved outcomes in children recently recovered from SAM potentially due to earlier identification of relapse to MAM or SAM or due to increased awareness of a child's health status.

## 2 Methods

### 2.1 Study Setting

This study was conducted in Boromo, Burkina Faso, which is located in central Burkina Faso. The study was implemented in 10 Centers de Santé et de Promotion Sociale (CSPS), which are primary healthcare facilities that provide outpatient primary treatment, preventative care, and maternity care. These facilities host outpatient nutritional programs once per week, during which they screen children for SAM and provide care for uncomplicated SAM. The standard outpatient nutritional program in Burkina Faso provides RUTF (a standard quantity based on weight at admission to the nutritional program, e.g., 15 sachets of Plumpy'nut for children 5–6.9 kg), antiparasitics, antibiotics, missing vaccinations, and malaria screening and treatment. Children in the outpatient nutritional programs are followed weekly until nutritional recovery. Children who have not recovered by 12 weeks after admission to the nutritional program are referred for inpatient care. After recovery, caregivers are requested to bring their children back to the clinic monthly for 3 months. Children with edematous malnutrition are treated on an inpatient basis. Supplemental food for moderate acute malnutrition (MAM) was not readily available in the study area at the time of the study. The MAM treatment program consisted of monthly screening, nutritional counseling, and cooking demonstrations for families to provide nutritious foods.

### 2.2 Study Design

This study was a 1:1 randomized controlled trial in which caregiver‐child dyads were randomized to either training in weekly at‐home screening using standard MUAC tapes or standard of care, which includes monthly, clinic‐based follow‐up visits. Dyads were followed for 6 months from enrollment. The trial was designed to evaluate both feasibility and clinical endpoints. The prespecified primary outcome was time to relapse detection.

### 2.3 Recruitment and Eligibility Criteria

Caregiver‐child dyads were recruited from the CSPS facilities that provided the outpatient nutritional program. Registers for the program were reviewed for children who recovered, and caregivers were screened for eligibility in the trial. Dyads were eligible to participate if the caregiver was at least 18 years of age, the child was between 6 and 59 months of age, the child had recovered from an episode of SAM per Burkinabè national guidelines (MUAC ≥ 12.5 cm and/or WHZ ≥ −2, depending on which criterion the child was admitted to the nutritional program) in the past month, the family was planning stay in the study area for 6 months to facilitate retention in the study, and with appropriate consent from the caregiver or guardian. Children who had recovered from a case of uncomplicated SAM defined as WHZ < −3 and/or MUAC < 11.5 cm in the past 30 days were included in the trial. Children who had experienced complicated SAM (including those who had kwashiorkor, which is treated on an inpatient basis in Burkina Faso) were not included.

### 2.4 Randomization and Masking

Caregiver‐child dyads were randomized on a 1:1 basis without stratification or blocking. Randomization was performed by the trial's data coordinating center using a computer‐based algorithm. Study identification numbers were linked to randomized study group assignment in the trial's mobile data collection application (CommCare, Dimagi, Inc). The randomization group was only revealed via the application after the caregiver‐child dyad was enrolled and baseline measurements were completed. Given the nature of the intervention, investigators, participants, and caregivers were not masked to their assigned treatment. Nutrition program staff who evaluate children returning to the clinic for relapse and outcome assessors were masked to the intervention assignment.

### 2.5 Baseline Assessment

At baseline, caregiver‐child dyads completed a quantitative survey assessing child, caregiver, and household‐level characteristics. These included clinical characteristics for the child (e.g. anthropometry and hemoglobin), the child's dietary diversity (if the child had eaten a series of 11 food groups in the past 6 days, summarized in a composite dietary diversity score (Sie et al. 2018)), the caregiver's education and occupation, and household food insecurity using the Household Food Insecurity Access Scale (Knueppel et al. 2009).

### 2.6 Interventions

Caregivers who were randomized to the MUAC screening arm were trained by a study team member after enrollment in the trial. Each caregiver randomized to MUAC screening was given a standard color‐coded MUAC tape and a plastic storage container with a lid for storing the tape. Caregivers received a brief training in how to use and interpret the MUAC tape. Caregivers were instructed to measure the child's MUAC weekly for 6 months and to return to the CSPS with their child for care if their child's MUAC measurement was red (SAM, MUAC < 11.5 cm) or yellow (MAM, MUAC < 12.5 cm). In the standard of care arm, caregivers were instructed to bring their child in monthly for screening and nutritional counseling for 3 months, per Burkinabè national guidelines. Regardless of their anthropometric measurements, children who recovered from SAM were enrolled in a MAM care program for 3 months. The MAM care program includes nutritional counseling for families and monthly screening for progression to SAM at the clinic for 3 months. Although to be recovered children do not meet criteria for MAM, this post‐recovery care was developed because children who have recently recovered from SAM are considered a particularly vulnerable group. Children in the caregiver screening arm received the same care packages provided in the standard of care.

### 2.7 Follow‐Up Procedures

Participants were followed monthly for 6 months (including the 3 months of standard of care follow‐up plus 3 additional months), and the primary outcome was at 6 months post‐enrollment. Per Burkinabè guidelines, caregivers were requested to bring their children back monthly for acute malnutrition screening and nutritional counseling for 3 months after enrollment. To understand adherence to the follow‐up schedule, no efforts beyond those routinely implemented by the nutritional program to follow up children were employed. The primary endpoint of the trial was 6 months after enrollment, at which point study staff contacted caregivers to bring their children to the clinic for the final follow‐up visit. At each study visit, anthropometric measurements were collected, including weight using a standing scale (Seca GmBH, Germany), height using a ShorrBoard (Weigh and Measure, LLC, Maryland, USA), and MUAC using a standard MUAC tape. Children who were too young to stand were weighed with their caregiver and had length rather than height measurements collected. At each follow‐up visit, caregivers were asked about any care they sought for their child, reasons for seeking healthcare, and hospitalization.

### 2.8 Primary Outcome

The primary prespecified clinical outcome was time to detection of relapse to either MAM or SAM. We also assessed the proportion of children who relapsed to either MAM or SAM by 6 months. WHZ < −3 and/or MUAC < 11.5 cm was considered a relapse to SAM, and WHZ < −2 and WHZ ≥ −3 and/or MUAC < 12.5 cm and MUAC ≥ 11.5 cm was considered relapse to MAM. Time to detection of relapse by the nutritional program was used as the primary outcome as the primary research question was whether MUAC screening decreased time to detection of relapse. Therefore, no midpoint imputation was used. Relapses were detected both through routine monthly follow‐up visits as well as through unscheduled visits to the clinic. A unique study identification number was placed in the government‐issued health card for each child (required for the child to access free governmental healthcare services), and this number was recorded in the medical record for all medical visits during the study period. Medical records were reviewed weekly and all unscheduled visits were recorded in the study's mobile data application, along with anthropometric measurements and whether the child had relapsed to either MAM or SAM. Children who relapsed to SAM were re‐admitted to the nutritional program and received the standard package of interventions described previously. Families of children with MAM received nutritional counseling and cooking demonstrations.

### 2.9 Secondary Outcomes

Secondary anthropometric endpoints included weight gain in g/kg/day, height change in mm/day, MUAC, WHZ, weight‐for‐age Z‐score (WAZ), and height‐for‐age Z‐score (HAZ) at 6 months. Z‐scores were based on 2006 WHO growth standards ($author1$ et al. 2006). Secondary clinical endpoints included seeking care for malnutrition as reported by the caregiver, if the child was hospitalized during the study period, vital status, and a combined endpoint assessing if the child was hospitalized and/or died during the study. In addition, we assessed whether the child had at least one clinic visit (for any reason) during the follow‐up period and measured hemoglobin at 6 months after enrollment (Hemocue Hb 301 System, Ängelholm, Sweden). Finally, we assessed adherence to the follow‐up scheduled by arm for each monthly visit per guidelines for post‐recovery surveillance.

### 2.10 Feasibility Endpoints

Feasibility endpoints included an assessment of accuracy of caregiver MUAC measurements versus a gold standard anthropometrist at baseline and 6 months and a caregiver acceptability survey at 6 months among caregivers of children in the MUAC screening arm. For the accuracy assessment, at baseline after completion of training, each caregiver in the MUAC screening arm was asked to measure the MUAC of a child other than their own and report the color (green, yellow, or red). The same child was independently measured by a nutrition staff member with extensive experience in measuring MUAC. This same procedure was repeated at the 6‐month visit (without a refresher training for the caregiver). For acceptability, at 6 months caregivers in the MUAC screening arm were asked to report how often they screened their child for malnutrition, the ease of screening, and were asked to bring their MUAC tapes and show them to study staff to assess the condition of the tape.

### 2.11 Sample Size

The sample size was based on feasibility of recruitment given the timeframe (recruitment over a single malnutrition season) and resources for this feasibility trial. Assuming a probability of relapse of 25% in the standard of care arm (25 events), we estimated that inclusion of 200 children recently recovered from SAM (*N* = 100 per arm) would yield at least 80% power to detect a hazard ratio for detection of relapse of 0.33.

### 2.12 Statistical Analysis

We compared time to detection of relapse using a log‐rank test with a term for randomized treatment assignment and calculated a hazard ratio to estimate differences in time to detection of relapse between groups. For probability of relapse, we compared the proportion of children with the outcome by study arm using a Fisher's exact test. Continuous anthropometric outcomes were compared by arm with a term for randomized treatment arm and a term for the baseline anthropometric measure for the MUAC, WHZ, WAZ, and HAZ outcomes. Adjustment for baseline values of continuous outcomes was done to reduce the variance in the effect estimate (Clifton and Clifton 2019). Acceptability and accuracy outcomes were analyzed descriptively. All analyses were completed in R (The R Studio for Statistical Computing, Vienna, Austria).

### 2.13 Ethics Statement

The study was reviewed and approved by the Institutional Review Board at the University of California, San Francisco (Protocol 23‐38829) and the Comité d'Ethique et Institutionnel at the Institut National de Santé Publique in Ouagadougou, Burkina Faso (Protocol 2023‐0000011). Written informed consent was obtained from the caregiver for both their participation and the participation of their child. The trial was registered at ClinicalTrials.gov (NCT05932992).

## 3 Results

**Figure 1:**
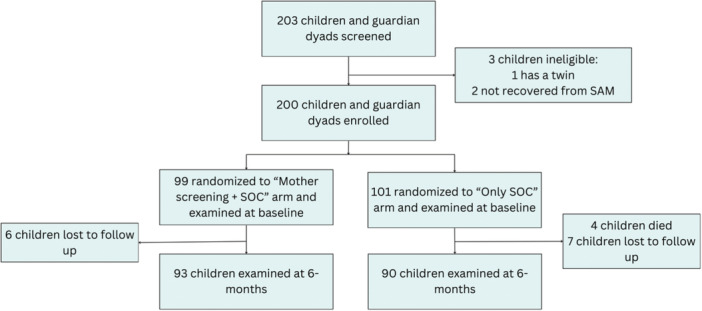
CONSORT flow diagram for children included in the study.

Of 203 caregiver‐child dyads screened for the trial, 200 met eligibility criteria and were enrolled from December 2023 until June 2024, with the final follow‐up visit completed in December 2024. Of these, 99 were randomized to the MUAC screening plus standard of care group (henceforth referred to as “MUAC screening group”) and 101 were randomized to the standard of care only group (henceforth referred to as “SOC group”). At the 6‐month visit, 93 (94%) were seen in the MUAC screening group (*N* = 6 lost to follow‐up), and 90 (89%) were seen in the SOC group (*N* = 7 lost to follow up and *N* = 4 died; Figure 1). Baseline child, caregiver, and household characteristics were similar between groups (Table 1). Approximately one‐third of children attended each monthly follow‐up visit in both study groups (Table 2), with no evidence of a difference in follow‐up visit attendance with the exception of the second month, when follow‐up visit attendance was modestly higher in the MUAC screening group compared to the SOC group.

**Table 1:** Baseline characteristics among caregiver‐child dyads by randomized treatment assignment.

	MUAC screening (*N* = 99)	Standard of care (*N* = 101)
Child characteristics		
Child's sex		
Male, *N* (%)	56 (56.6%)	48 (47.5%)
Female, *N* (%)	43 (43.4%)	53 (52.5%)
Age in months, median (IQR)	14 (7.5)	15 (12)
Admission criterion to nutritional program		
WHZ only, *N* (%)	13 (13.1%)	10 (9.9%)
MUAC only, *N* (%)	23 (23.2%)	20 (19.8%)
WHZ and MUAC, *N* (%)	63 (63.6%)	71 (70.3%)
MUAC at enrollment, median (IQR)	13 (0.4)	13.1 (0.6)
WHZ at enrollment, mean (SD)	−1.5 (0.8)	−1.6 (0.8)
WAZ at enrollment, mean (SD)	−2.1 (0.8)	−2.1 (0.9)
HAZ at enrollment, mean (SD)	−1.9 (1.5)	−1.8 (1.4)
Hemoglobin, g/dL, median (IQR)	10.2 (1.3)	10.4 (1.7)
Dietary diversity score, median (IQR)	11 (3)	10 (4)
Caregiver characteristics		
Caregiver relationship		
Mother, *N* (%)	98 (99.0%)	98 (97.0%)
Grandmother, *N* (%)	1 (1.0%)	3 (3.0%)
Caregiver age, years, median (IQR)	24 (10)	26 (8)
Caregiver education		
None, *N* (%)	70 (70.7%)	82 (81.2%)
Primary, *N* (%)	21 (21.2%)	11 (10.9%)
Secondary, *N* (%)	5 (5.1%)	6 (6.0%)
College or above, *N* (%)	3 (3.0%)	2 (2.0%)
Caregiver occupation		
Household, *N* (%)	85 (85.9%)	93 (92.1%)
Agriculture, *N* (%)	9 (9.1%)	5 (5.0%)
Retail, *N* (%)	3 (3.0%)	3 (3.0%)
Other, *N* (%)	2 (2.0%)	0 (0%)
Household characteristics		
Number of children < 5 years in household, median (IQR)	2 (2)	2 (2)
Household food insecurity		
Food secure, *N* (%)	39 (39.4%)	34 (33.7%)
Mildly food insecure, *N* (%)	18 (18.2%)	25 (24.8%)
Moderately food insecure, *N* (%)	26 (26.3%)	21 (20.8%)
Severely food insecure, *N* (%)	16 (16.2%)	21 (20.8%)
Water source		
Running water or tubewell, *N* (%)	53 (53.5%)	53 (52.5%)
Protected dug well, *N* (%)	44 (44.4%)	47 (46.5%)
Unprotected dug well, *N* (%)	1 (1.0%)	1 (1.0%)
Sanitary installation		
Improved pit latrine, *N* (%)	15 (15.2%)	13 (12.9%)
Simple latrine with slab, *N* (%)	78 (78.8%)	83 (82.2%)
Simple sand dale latrine, *N* (%)	6 (6.1%)	5 (5.0%)

**Table 2:** Follow‐up visit attendance by randomized study arm for the first 3 months post‐recovery.

	MUAC screening (*N *= 99)	Standard of care (*N *= 101)
Month 1, *N* (%)	38 (38.4%)	34 (33.7%)
Month 2, *N* (%)	39 (39.4%)	25 (24.8%)
Month 3, *N* (%)	31 (31.3%)	34 (33.7%)

The primary prespecified outcome, time to detection of relapse to SAM or MAM, was not statistically significantly different in the MUAC screening group compared to the SOC group (hazards ratio, HR, 0.65, 95% CI 0.38 to 1.12; Table 3, Supporting Information: Figure 1). There was no evidence of a difference in time to detection of relapse for SAM (Table 3). There was a 45% lower hazard of relapse to MAM in the MUAC group compared to SOC group (HR 0.55, 95% CI 0.31 to 0.99, Table 3). The hazard of relapse was similar for the first 90 days following enrollment in the trial, with most relapses occurring after this timepoint and more frequently in the SOC arm compared to the MUAC screening arm (Supporting Information: Figure 1). Because fewer than 50% of enrolled children relapsed, calculation of median time to relapse in days was not possible.

**Table 3:** Relapse, anthropometric, and clinical outcomes at 6 months by randomized study arm.

	MUAC screening	Standard of care	Mean difference or HR/RR (95% CI)
Number of relapses to MAM or SAM	22 (23.2%)	33 (34.4%)	0.67 (0.43, 1.07)
Hazard of relapse to MAM or SAM	49 (115)	103 (139)	0.65 (0.38 to 1.12)
Number of relapses to SAM	6 (6.3%)	5 (5.2%)	1.21 (0.38 to 3.84)
Hazard of relapse, SAM			1.22 (0.37 to 4.00)
Number of relapses to MAM	16 (16.8%)	28 (29.2%)	0.58 (0.34 to 0.99)
Hazard of relapse, MAM			0.55 (0.31 to 0.99)
Anthropometric outcomes			
Growth, g/kg/day (SD)	1.12 (0.8)	1.02 (0.8)	0.10 (−0.14 to 0.34)
Height change, mm/day (SD)	0.23 (0.2)	0.19 (0.36)	0.04 (−0.04 to 0.12)
MUAC, mean (SD)	13.7 (0.6)	13.7 (0.8)	0.10 (−0.07 to 0.28)
WHZ, mean (SD)	−0.52 (1.3)	−0.74 (1.4)	0.23 (−0.18 to 0.63)
WAZ, mean (SD)	−1.48 (1.1)	−1.71 (1.1)	0.22 (−0.10 to 0.53)
HAZ, mean (SD)	−2.17 (1.5)	−2.28 (1.4)	0.16 (−0.25 to 0.56)
Clinical outcomes			
Sought care for malnutrition^1^ (RR)	13 (14.0%)	14 (15.6%)	0.90 (0.45 to 1.80)
Hospitalized (RR)	3 (3.2%)	9 (10.0%)	0.32 (0.09 to 1.15)
Died (RR)	0 (0%)	4 (4.3%)	NA
Hospitalized and/or died (RR)	3 (3.2%)	13 (13.8%)	0.23 (0.07 to 0.79)
At least one clinic visit during follow‐up (RR)	68 (73.1%)	61 (64.9%)	1.13(0.93 to 1.37)
Hemoglobin, g/dL, mean (SD)	10.2 (1.0)	10.2 (1.2)	0.009 (0.33 to 0.31)

Abbreviations: HR, hazards ratio; HAZ, height‐for‐age Z‐score; IQR, interquartile range; MUAC, mid‐upper arm circumference; MAM, moderate acute malnutrition; RR, risk ratio; WHZ, weight‐for‐height Z‐score; (defined as WHZ < −2 & ≥ −3 and/or MUAC < 12.5 cm & ≥ 11.5 cm); SAM, severe acute malnutrition (defined as WHZ < −3 and/or MUAC < 11.5 cm); SD, standard deviation; WAZ, weight‐for‐age Z‐score; 1As measured by caregiver self‐report.

We also assessed cumulative incidence of relapse. A total of 22 (23.2%) relapses to SAM or MAM were recorded in the MUAC screening group and 33 (34.4%) in the SOC group, corresponding to non‐statistically significant 33% lower probability of identification of relapse to MAM or SAM over the 6‐month period after recovery (risk ratio, RR, 0.67, 95% confidence interval, CI, 0.43–1.07; Table 3). Relapse to SAM was relatively uncommon (6.3% in the MUAC screening group and 5.2% in the SOC group), and differences were largely driven by relapses to MAM (Table 3). The probability of relapse to MAM was statistically significantly lower in the MUAC screening group compared to the SOC group (RR 0.58, 95% CI, 0.34–0.99, Table 3).

There were no statistically significant differences in anthropometric endpoints by study group. Both WHZ and WAZ were higher in the MUAC screening group compared to the SOC group, but these differences were not statistically significantly different (Table 3). Results were similar after adjusting for baseline (Supporting Information: Table 1). Four deaths occurred over the course of the study, all of which were in the SOC group. The combined endpoint hospitalization and/or death was statistically significantly lower in the MUAC only screening group compared to the SOC only group, with a 77% reduction in hospitalization and/or mortality in those in the MUAC screening group (RR 0.23, 95% CI, 0.07–0.79; Table 3).

Almost all children included in the MUAC screening accuracy assessment were in the “green” zone of the MUAC tape, and concordance between the caregiver and the gold standard was 100% (Supporting Information: Table 2). Overall, acceptability of MUAC screening was high, with approximately half of caregivers reporting screening their children at least once a week with the MUAC tape, and most reporting screening at least monthly (Table 4). Approximately 85% of caregivers reported that screening was very or somewhat easy, and approximately 80% produced a MUAC tape at the 6 months visit that was in good condition (Table 4).

**Table 4:** Acceptability endpoints at 6 months (MUAC screening arm only).

Characteristic	*N* (%)
How often did you screen your child for malnutrition?	
At least once/week	48 (51.6%)
Every other week	9 (9.7%)
Monthly	21 (22.6%)
Less than monthly	4 (4.3%)
Never	11 (11.8%)
How easy was it to screen your child for malnutrition?	
Very easy	34 (36.6%)
Somewhat easy	45 (48.4%)
Neither easy nor difficult	3 (3.2%)
Somewhat difficult	11 (11.8%)
Very difficult	0 (0%)
Condition of the MUAC tape	
Tape not available	9 (9.7%)
Good condition	75 (80.6%)
Some wear and tear	8 (8.6%)
Not usable	1 (1.1%)

## 4 Discussion

In this study, the rate of relapse was non‐significantly lower among children who had caregivers who were trained to use simple MUAC tapes to screen them for signs of relapse. These children also had reduced risk of hospitalization and/or death, and non‐significantly improved weight‐based anthropometric measurement. These results suggest that MUAC tapes may be an inexpensive and accessible intervention to improve outcomes in this vulnerable group of children. While caregiver self‐report of care seeking for malnutrition did not significantly differ between arms, time to detection of relapse was shorter in the MUAC screening arm, suggesting that cases of relapse were identified earlier. We hypothesized that equipping caregivers with MUAC tapes could lead to earlier detection of relapse if it occurs and overall lower probability of relapse if caregivers are more aware of their child's nutritional status. These results are consistent with our hypothesis that equipping caregivers of children who recently recovered from SAM may lead to improved clinical outcomes for children, suggesting a fully powered study evaluating the role of family MUAC screening following recovery from SAM is warranted.

Previous work has demonstrated that mothers can accurately use MUAC tapes to screen their children for acute malnutrition (Blackwell et al. 2015; Alé et al. 2016; Tamirat et al. 2025). In the setting of SAM treatment, a reduced follow‐up schedule that included training caregivers to complete MUAC measurements and assess for clinical danger signs found that the reduced follow‐up schedule was inferior to the standard weekly schedule for nutritional recovery, but the rate of default from the program was lower in the monthly follow‐up group (Hitchings et al. 2022). Relapse at 3 months was less common in the monthly follow‐up group compared to the weekly follow‐up group that did not include caregiver training. Equipping caregivers with a tool and knowledge related to their child's care may empower them to be more actively engaged in and aware of their child's health status, and seek care earlier, theoretically leading to better outcomes for their child. For children who have recovered and for whom weekly follow‐up is not available, caregiver screening may lead to increased awareness of the child's health status. In the present study, children in the caregiver screening arm had reduced risk of hospitalization and death compared to the standard of care group, which may support the hypothesis caregivers who were more engaged in their child's post‐recovery follow‐up had increased awareness of their child's health that led to earlier intervention and improved outcomes. While our data suggested that MUAC screening improved growth and clinical outcomes for children, most results were not statistically significantly different, and larger studies are needed to confirm these findings.

Our results are in line with recent estimates from Mali suggesting that approximately 30% of children relapse to MAM or SAM in the 6‐month period following discharge from a community‐based management of SAM program (King et al. 2025). As expected, the probability of relapse increased over time with the probability of relapse increasing at approximately 30 days after enrollment in the trial. This timing is likely due to time to development and detection of relapse. SAM treatment programs in many settings are under‐resourced, and global interruptions in foreign aid may further weaken nutritional programming (Locks et al. 2025; Anfaal et al. 2025). Interventions that reduce the risk of relapse will reduce the number of children who are re‐admitted to nutritional programs and may reduce some stress on these systems. While current guidelines include clinic‐based surveillance and in some settings provision of supplemental food to prevent relapse, interventions to improve outcomes in these children may be especially important in the context of global food supply interruptions for the treatment and prevention of acute malnutrition. MUAC tapes are inexpensive (<$1 per tape) and thus scaling of family MUAC interventions for relapse detection may be economically feasible. In this study, the use of MUAC tapes was acceptable to caregivers, and most caregivers indicated that it was easy to screen their child, and they screened at least monthly. MUAC tapes were in good condition at the 6‐month visit, which may indicate that caregivers can maintain them for many months when provided a storage container. However, it is possible that the good condition of the tapes indicates they were not being routinely used. Future work could include structured observation or other qualitative assessment of the use of MUAC tapes in this context.

This study must be considered in the context of several limitations. First, the study was designed as a pilot and feasibility trial for integrating caregiver MUAC screening into post‐recovery treatment protocols, and thus the study was not powered for many clinically important outcomes. For example, children in the MUAC screening group had more than 0.20 z‐score improvement in WHZ and WAZ compared to the SOC group, which are considered relatively large improvements in these measures, but results were not statistically significantly different. Studies with larger sample sizes that are adequately powered for important clinical outcomes, including relapse and growth, are needed to confirm these findings. However, this study did demonstrate a large, statistically significant reduction in hospitalization and/or mortality, and results were consistently in favor of the MUAC screening intervention across a range of outcomes. By design, follow‐up was low during monthly visits following enrollment. This design choice was made to allow for more accurate assessment of real‐world adherence to the follow‐up schedule, which was hypothesized to be low at the beginning of the study. Follow‐up was high at the 6‐month primary outcome visit. However, the low frequency of monthly follow‐up visits may have meant that some relapses were missed, if for example a child relapsed and recovered outside on their own outside of seeking medical care. Our estimates of relapse could be an underestimate of the true risk of relapse. Potentially eligible children were identified on study registers to facilitate recruitment, which could have led to some variability in time since recovery at the time of enrollment. However, we limited eligibility to children who had recovered within the past 30 days (e.g. before their first scheduled post‐recovery follow‐up visit). The accuracy assessment for assessing caregiver accuracy in measuring MUAC was limited by the fact that most children included in this assessment were not malnourished. Additional studies evaluating the accuracy of caregiver MUAC screening included a range of nutritional statuses and over time is warranted. Finally, children who relapsed to SAM and MAM were referred to care programs for acute malnutrition. For SAM in particular, this involved re‐admission to the nutritional program and receipt of RUTF and other components of the program that promote growth. Anthropometric endpoints at 6 months therefore include effects of receipt of treatment for SAM and MAM and thus may be biased towards the null if children who received treatment for acute malnutrition had substantial catch‐up growth. However, there were relatively few SAM cases, and MAM treatment is inconsistent and often does not include supplemental food, so it is unlikely this had a major impact on outcomes.

In conclusion, the results of this study suggest that equipping and training caregivers of children receiving recovered from SAM with a MUAC tape is acceptable and feasible and may improve outcomes for children compared to standard of care in Burkina Faso. The results of this study support larger‐scale effectiveness trials in similar settings to evaluate the impact of family MUAC on post‐recovery outcomes in children with SAM. While this study was underpowered for most clinical outcomes, results were consistent in that they suggested improvement in children whose caregivers had been trained to screen them with the MUAC tape. Given that MUAC tapes are inexpensive and safe to use, this may be an effective intervention to improve outcomes during the vulnerable post‐recovery period for children who have had SAM.

## Author Contributions

M.B, C.D, B.F.A, A.S, and C.E.O designed the study. M.B, C.D, M.O, F.Z, I.K, E.L, J.L.M, H.B, A.S, and C.E.O supervised data collection. I.K and I.F were responsible for data management. I.K, I.F, and B.F.A analyzed the data. All authors interpreted data. C.E.O wrote the paper. All authors critically reviewed and edited the paper.

## Conflicts of Interest

The authors declare no conflicts of interest.

## Supporting information

MAMAN Pilot Supplement R1.

## Data Availability

Data are available upon reasonable request from the corresponding author.
