# Supplementary material for: Caregiver Screening for Relapse Among Children Recently Recovered From Severe Acute Malnutrition: A Randomized Controlled Feasibility Trial
Source: Matern Child Nutr. 2026 Jan 22;22(1):e70160. doi: 10.1111/mcn.70160 (PMC12826110; doi:10.1111/mcn.70160)

**Supplemental Table 1.** Anthropometric outcomes at 6 months adjusting for baseline measures

|  | **MUAC Screening** | **Standard of Care** | **Mean Difference (95% CI)** | **P-value** |
| --- | --- | --- | --- | --- |
| MUAC, mean (SD) | 13.7 (0.6) | 13.7 (0.8) | 0.10  (-0.07 to 0.28) | 0.44 |
| WHZ, mean (SD) | -0.52 (1.3) | -0.74 (1.4) | 0.23  (-0.18 to 0.63) | 0.22 |
| WAZ, mean (SD) | -1.48 (1.1) | -1.71 (1.1) | 0.22  (-0.10 to 0.53) | 0.18 |
| HAZ, mean (SD) | -2.17 (1.5) | -2.28 (1.4) | 0.16  (-0.25 to 0.56) | 0.67 |

**Supplemental Table 2.** Mid-upper arm circumference (MUAC) measurement accuracy of caregivers compared to gold standard grader at baseline and 6 months (MUAC screening arm only)

|  |  | Gold Standard Grader | | |
| --- | --- | --- | --- | --- |
|  |  | Green | Yellow | Red |
| **Baseline** | | | | |
| Caregiver | Green | 91 | 0 | 0 |
|  | Yellow | 0 | 2 | 0 |
|  | Red | 0 | 0 | 0 |
| **6 months** | | | | |
| Caregiver | Green | 86 | 0 | 0 |
|  | Yellow | 0 | 0 | 0 |
|  | Red | 0 | 0 | 0 |

**Supplemental Figure 1.** Kaplan-Meier curve for time to relapse to moderate acute malnutrition (MAM) or severe acute malnutrition (SAM) by randomized study group (standard of care, SOC, only or mid-upper arm circumference, MUAC, screening plus SOC).


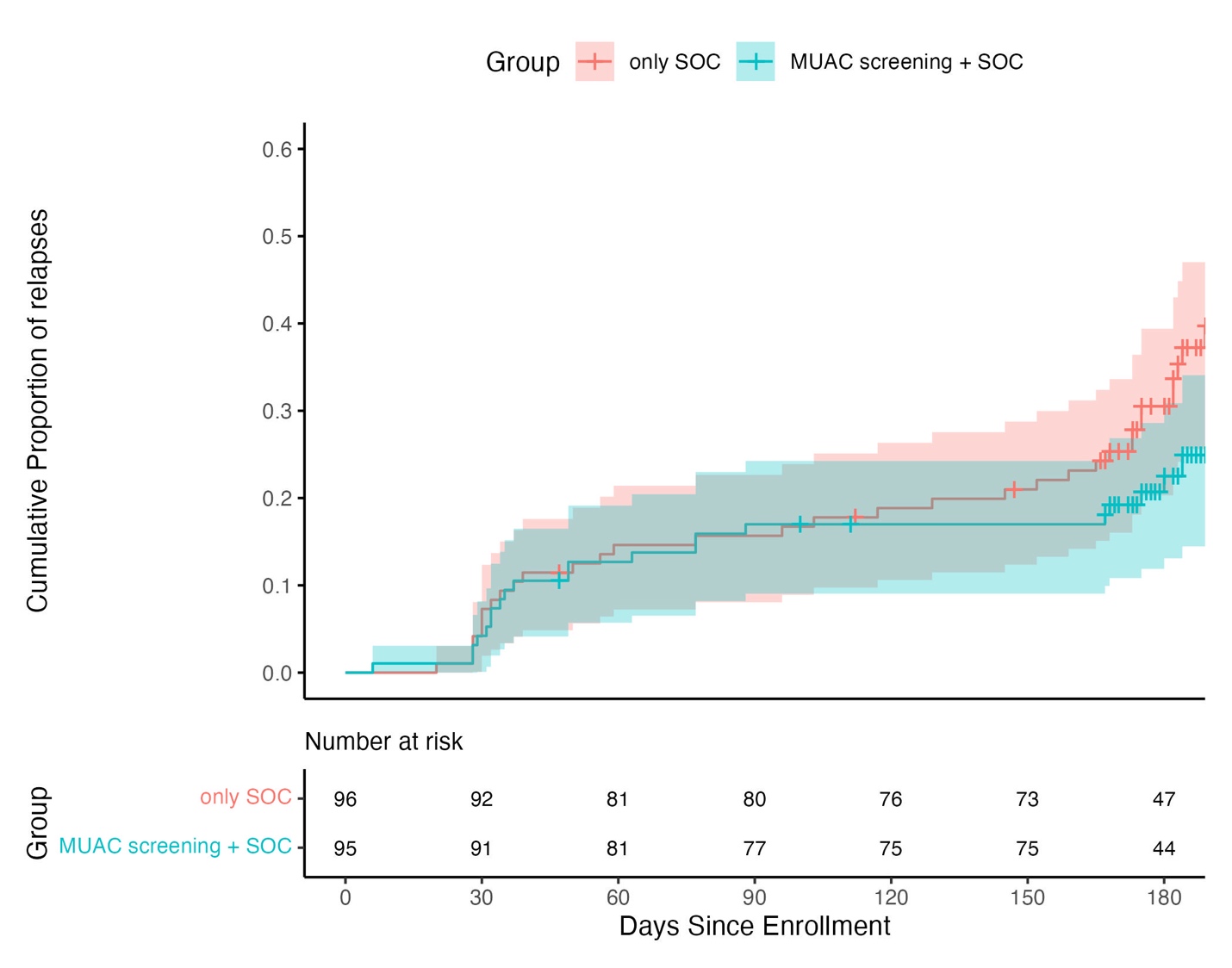

Supplement: Supplementary file 1 — MAMAN Pilot Supplement R1. [file MCN-22-e70160-s001.docx]
